# Fragmented QRS is associated with intraventricular dyssynchrony and independently predicts nonresponse to cardiac resynchronization therapy—Systematic review and meta‐analysis

**DOI:** 10.1111/anec.12750

**Published:** 2020-03-18

**Authors:** Raymond Pranata, Emir Yonas, Rachel Vania, Alexander Edo Tondas, Yoga Yuniadi

**Affiliations:** ^1^ Faculty of Medicine Universitas Pelita Harapan Tangerang Indonesia; ^2^ Faculty of Medicine Universitas YARSI Jakarta Indonesia; ^3^ Department of Cardiology and Vascular Medicine Faculty of Medicine Universitas Sriwijaya Dr. Mohammad Hoesin General Hospital Palembang Indonesia; ^4^ Department of Cardiology and Vascular Medicine Faculty of Medicine Universitas Indonesia National Cardiovascular Center Harapan Kita Jakarta Indonesia

**Keywords:** cardiac resynchronization therapy, fragmented QRS, heart failure, intraventricular dyssynchrony, nonresponse

## Abstract

**Background:**

Fragmented QRS (fQRS) is postulated to be associated with ventricular dyssynchrony and might be able to predict a nonresponse to cardiac resynchronization therapy (CRT) implantation. In this systematic review and meta‐analysis, we aim to assess whether fQRS can be a marker of intraventricular dyssynchronies in patients with ischemic and nonischemic cardiomyopathy and whether it is an independent predictor of nonresponse in patients receiving CRT.

**Methods:**

We performed a comprehensive search on topics that assesses fQRS and its association with intraventricular dyssynchrony and nonresponse to CRT up until September 2019.

**Results:**

Fragmented QRS is associated with intraventricular dyssynchrony (OR 10.34 [3.39, 31.54], *p* < .001; *I* ^2^: 80% with sensitivity 76.8%, specificity 77%, LR+ 3.3, and LR− 0.3). Subgroup analysis showed that fQRS is associated with intraventricular dyssynchrony in patients with narrow QRS complex (OR 20.92 [12.24, 35.73], *p* < .001; *I* ^2^: 0%) and nonischemic cardiomyopathy (OR of 19.97 [12.12, 32.92], *p* < .001; *I* ^2^: 0%). Fragmented QRS was also associated with a higher time‐to‐peak myocardial sustained systolic (Ts‐*SD*) (OR 15.19 [12.58, 17.80], *p* < .001; *I* ^2^: 0% and positive Yu index (OR 15.61 [9.07, 26.86], *p* < .001; *I* ^2^: 0%). Fragmented QRS has a pooled adjusted OR of OR of 1.70 [1.35, 2.14], *p* < .001; *I* ^2^: 62% for association with a nonresponse to CRT. QRS duration is found to be higher in nonresponders group mean difference −8.54 [−13.38, −3.70], *p* < .001; *I* ^2^: 70%.

**Conclusion:**

Fragmented QRS is associated with intraventricular dyssynchrony and is independently associated with nonresponse to cardiac resynchronization therapy.

## 1 INTRODUCTION

Fragmented QRS (fQRS) is often caused by myocardial scarring with consequent heterogeneous ventricular activation and dyssynchronous contraction of the ventricles.(Basaran et al., 2011) Several studies have shown that fQRS is commonly generated after myocardial infarction and reflects the varying conduction abnormalities and the delay of peri‐infarct area conductions due to myocardial necrosis or scarring. Fragmented QRS has been shown to appear 24–48 hr after onset of ACS symptoms.(Das, Khan, Jacob, Kumar, & Mahenthiran, 2006) It has been previously shown that increased fragmentation of QRS wave was associated with previous myocardial infarction, ventricular enlargement, and decreased LVEF.(Sha et al., 2011).

Cardiac resynchronization therapy (CRT) is an integral mode of intervention in patients with heart failure (HF) with reduced ejection fraction and was associated with reduced morbidity and mortality in appropriate patients.(Ponikowski et al., 2016; Yancy et al., 2017) Usually these patients have wide QRS, and the guidelines also adhere to the QRS duration. However, studies have shown that up to 50% of patients with HF and normal QRS duration has mechanical ventricular dyssynchrony, and these patients may benefit from CRT (Dohi, Suffoletto, Murali, Bazaz, & Gorcsan, 2005; Yu et al., 2006). There is the need for other methods of assessment other than QRS duration, although whether the benefit of CRT in normal QRS duration with dyssynchrony is not the scope of our study. Nonresponsiveness to CRT is also problematic; approximately 30% of those implanted with CRT did not respond well to the treatment (Bax et al., 2004; Yu et al., 2005). This leads to a costly procedure without apparent benefit and increased risk of other complications related to CRT.

Fragmented QRS is postulated to be one of the markers to fill these gaps in selective patients; it was thought to be associated with ventricular dyssynchrony and might be able to predict a nonresponse to CRT implantation. In this systematic review and meta‐analysis, we aim to assess (a) fQRS as a marker of intraventricular dyssynchronies in patients with ischemic and nonischemic cardiomyopathy and (b) whether fQRS is an independent predictor of nonresponse in patients receiving CRT.

## 2 METHODS

### 2.1 Search strategy

**Figure 1:**
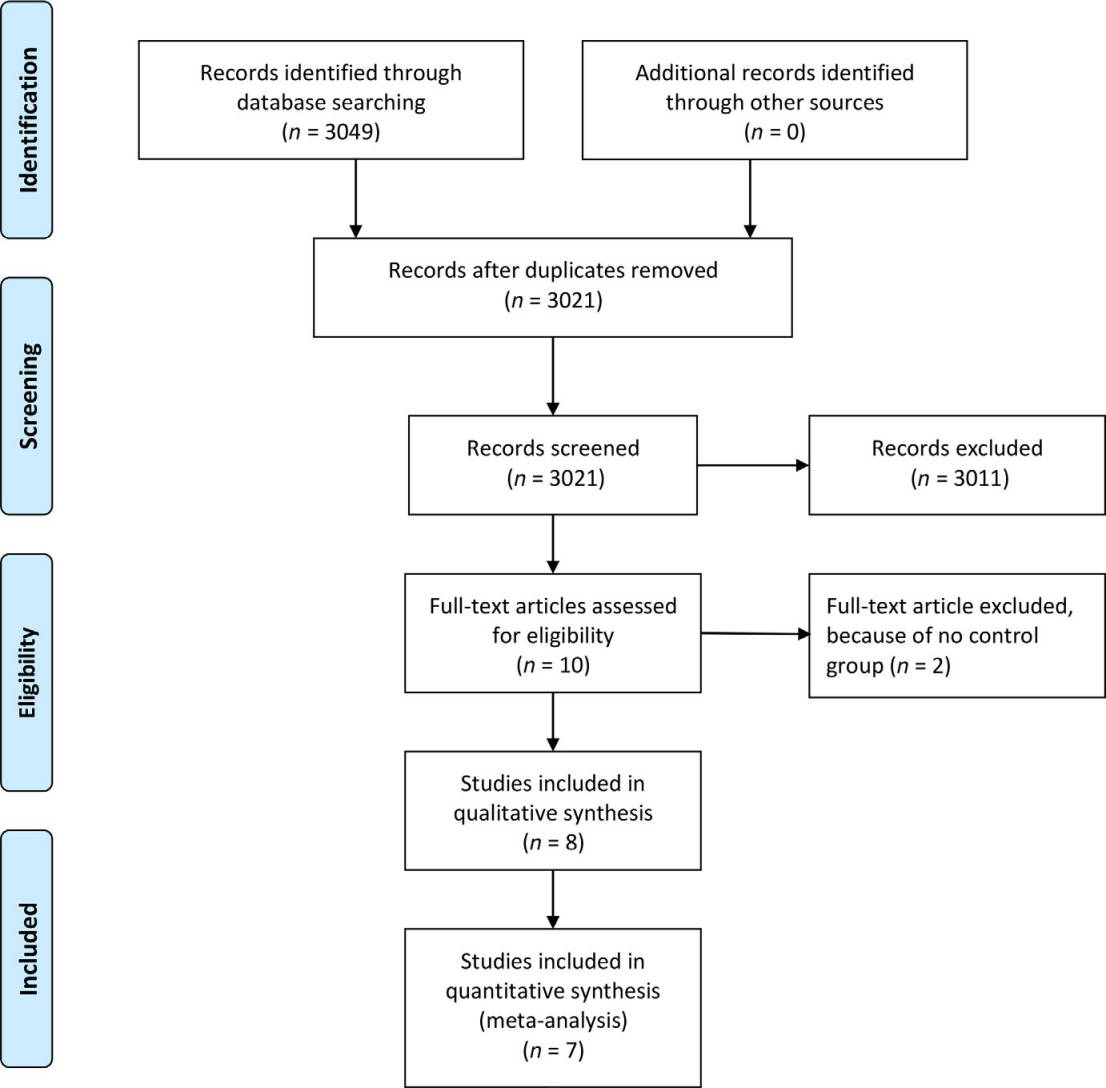
Study flow diagram

We performed a comprehensive search on topics that assesses fQRS and its association with intraventricular dyssynchrony and nonresponse to CRT with keywords (“fragmented QRS” and “dyssynchrony” OR “cardiac resynchronization therapy”) and its synonym from inception up until September 2019 through PubMed, Europe PMC, Cochrane Central Database, and hand sampling from potential articles cited by other studies. The records were then systematically evaluated using inclusion and exclusion criteria. We also perform hand sampling from references of the included studies. Two researchers (A.E.T and R.V) independently performed an initial search, and discrepancies were resolved by discussion. A Preferred Reporting Items for Systematic Reviews and Meta‐Analyses flowchart of the literature search strategy of studies was presented in Figure 1.

### 2.2 Selection criteria

The inclusion criteria for this study are all studies that assess fQRS, intraventricular dyssynchrony, and nonresponse to CRT. We include all related clinical researches/original articles and exclude case reports, review articles, and non‐English language articles.

### 2.3 Data extraction

Data extraction and quality assessment were done by two independent authors (E.Y and R.P) using standardized extraction form which includes authors, year of publication, study design, subject characteristics, study definition of dyssynchrony and nonresponse to CRT, sample size, QRS duration, prevalence of fQRS, and follow‐up duration.

### 2.4 Statistical analysis

To perform the meta‐analysis, we used RevMan version 5.3 software (Cochrane Collaboration) and STATA/MP 14 (StataCorp LLC). We used the odds ratio (OR) and a 95% CI as a pooled measure for dichotomous data. We used mean difference and its standard deviation (*SD*) as a pooled measure for the continuous data. Inconsistency index (*I* ^2^) test, which ranges from 0% to 100%, was used to assess heterogeneity across studies. A value above 50% or *p* < .05 indicates statistically significant heterogeneity. We used the Mantel–Haenzsel or inverse variance (for adjusted OR) method for OR and inverse variance for mean difference with a fixed‐effect model for meta‐analysis, and a random‐effect model was used in case of heterogeneity if appropriate. All *p* values were two‐tailed with a statistical significance set at .05 or below. We also perform sensitivity analysis and subgroup analysis whenever possible/appropriate.

## 3 RESULTS

We found a total of 3,049 results on the initial search. There were 3,021 records after removal of duplicates. A total of 3,011 records were excluded after screening the title/abstracts. After assessing ten full texts for eligibility, we excluded two because of no control group. We included eight studies in qualitative synthesis and seven studies meta‐analysis (Celikyurt et al., 2013, 2012; Hu et al., 2019; Rad et al., 2015; Sinha et al., 2016; Tigen et al., 2009; Yusuf et al., 2013; Zhao et al., 2015) (Figure 1). One study was excluded from meta‐analysis because it has different definition of nonresponder outcome that being “Cardiac death, heart transplantation, or HF hospitalization.” Four studies were prospective cohorts, three were cross‐sectional, and one was retrospective cohort. There were a total of 864 subjects from eight studies. (Table 1).

**Table 1:** Studies included in the systematic review

Study	Design	Patient characteristics	Definition of dyssynchrony	Definition of nonresponse	Samples	Nonischemic CM	QRS duration (ms)	QRS duration (fQRS vs. non‐fQRS)	fQRS+	Follow‐up
Hu et al., 2019	Retrospective Cohort	Reduced LVEF who successfully underwent CRT implantation	N/A	Cardiac death, heart transplantation, or HF hospitalization during 1‐year follow‐up	296	81.4%	166.7 ± 18.5	N/A	61 (20.6%)	12 months
Sinha et al., 2016	Cross‐sectional	Chronic HF due to nonischemic DCM, LVEF ≤ 35% and normal sinus rhythm having narrow QRS complexes (≤ 120 ms)	Ts‐*SD* > 32.6 ms	N/A	226	100%	96.7 ± 15	102.42 ± 14.05/91.10 ± 13.75	112 (49.5%)	N/A
Zhao et al., 2015	Prospective Cohort	Idiopathic DCM	time in peak anteroseptal wall to posterior wall strain > 130 ms or longitudinal strain delay index > 25%	N/A	49	100%	100.7 ± 8.5	101.5 ± 9.3/98.6 ± 6.7	20 (40.8%)	24 months
Rad et al., 2015	Prospective Cohort	HF undergoing CRT implantation	N/A	<15% decrease in LVESV at follow‐up	65	9%	139.5 ± 12.7	142.6 ± 13.5/137.4 ± 11.8	27 (41.5)	6 months
Celikyurt et al., 2013	Prospective Cohort	HF undergoing CRT implantation	N/A	<15% decrease in LVESV at follow‐up	105	61%	146 ± 18	N/A	48 (46%)	6 months
Yusuf et al., 2013	Prospective Cohort	Chronic HF due to nonischemic DCM, LVEF < 35% and normal sinus rhythm having narrow QRS complexes (< 120 ms)	Ts‐*SD* > 32.6 ms	N/A	100	100%	94.8 ± 14.1	99.42 ± 13.05/90.10 ± 13.75	50 (50%)	N/A
Celikyurt et al., 2012	Prospective Cohort	HF with a wide QRS complex undergoing CRT implantation	IVMD ≥ 40 ms and tissue Doppler velocity opposing‐wall delay ≥ 65 ms	<15% decrease in LVESV at follow‐up	53	63.8%	139.6 ± 15.9	143 ± 13/138 ± 17	17 (32.1%)	6 months
Tigen et al., 2009	Cross‐sectional	Nonischemic DCM with sinus rhythm and narrow QRS	Max‐ASE Sys and Max‐ASE Dias of more than 100 ms, and Max‐ASE to Mean Sys of more than 60 ms	N/A	60	100%	N/A (Narrow)	N/A	20 (33.3%)	N/A

Abbreviations: CRT, cardiac resynchronization therapy; DCM, dilated cardiomyopathy; HF, heart failure; IVMD, interventricular mechanical delay; LVEF, left ventricular ejection fraction; N/A, not assessed/available/applicable; Ts‐*SD*, time‐to‐peak myocardial sustained systolic and its standard deviation.

### 3.1 Characteristics of the study

Some of the studies enrolled patients with a narrow QRS complex exclusively. There are four studies that enroll nonischemic cardiomyopathy only, and there are four studies that enroll ischemic and nonischemic cardiomyopathy. The definition of dyssynchrony varied across studies. Cohorts have follow‐up ranging from 6 months to 24 months.

### 3.2 Fragmented QRS and intraventricular dyssynchrony

**Figure 2:**
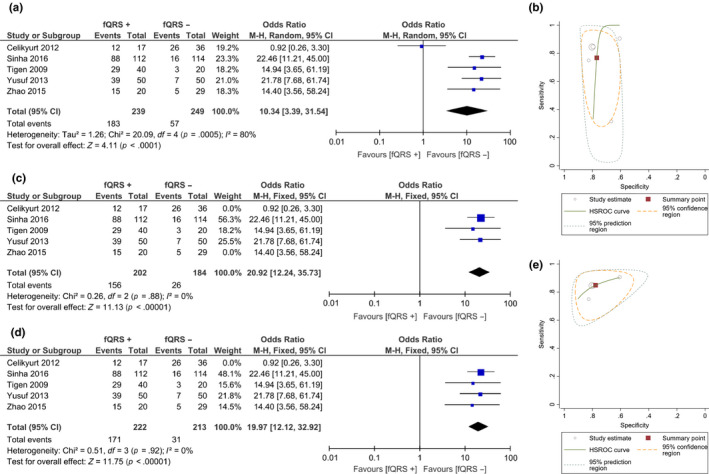
Fragmented QRS and intraventricular dyssynchrony. Fragmented QRS was associated with intraventricular dyssynchrony (a) and has sensitivity 76.8% and specificity 77% (b). Fragmented QRS was associated with intraventricular dyssynchrony upon subgroup analysis on patients with narrow QRS complex (c) and nonischemic cardiomyopathy (d). Nonischemic cardiomyopathy subgroup has sensitivity 84.8% and specificity 77.7% (e)

Fragmented QRS has a pooled OR of 10.34 [3.39, 31.54], *p* < .001; *I* ^2^: 80%, *p* < .001 (Figure 2a) for intraventricular dyssynchrony. Fragmented QRS has a sensitivity 76.8%, specificity 77%, LR + 3.3, and LR‐ 0.3 for predicting intraventricular dyssynchrony (Figure 2b). Upon sensitivity analysis by removing Celikyurt et al. (2012 study, pooled OR of 19.97 [12.12, 32.92], *p* < .001; *I* ^2^: 0%, *p* = .92.

Subgroup analysis on patients with narrow QRS complex showed that fQRS was associated with intraventricular dyssynchrony (OR 20.92 [12.24, 35.73], *p* < .001; *I* ^2^: 0%, *p* = .88) (Figure 2c). Subgroup analysis on nonischemic cardiomyopathy showed an OR of 19.97 [12.12, 32.92], *p* < .001; *I* ^2^: 0%, *p* = .92 (Figure 2d); and sensitivity 84.8%, specificity 77.7%, LR+ 3.82, and LR− 0.19 (Figure 2e).

Fragmented QRS was also associated with a higher time‐to‐peak myocardial sustained systolic and its standard deviation (Ts‐*SD*) (OR 15.19 [12.58, 17.80], *p* < .001; *I* ^2^: 0%, *p* = 1) and positive Yu index (OR 15.61 [9.07, 26.86], *p* < .001; *I* ^2^: 0%, *p* = .46).

### 3.3 Fragmented QRS and nonresponse to cardiac resynchronization therapy

**Figure 3:**
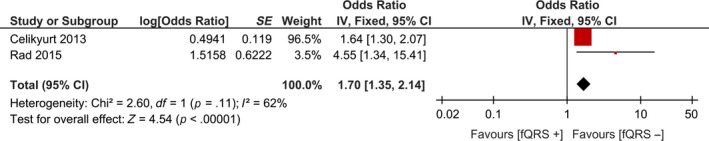
Fragmented QRS and nonresponse to cardiac resynchronization therapy. Fragmented QRS was associated with nonresponse to cardiac resynchronization therapy

Fragmented QRS has a pooled adjusted OR of 1.70 [1.35, 2.14], *p* < .001; *I* ^2^: 62%, *p* = .11 for association with a nonresponse to CRT (Figure 3). Hu et al. have a different definition of nonresponse to CRT that being “Cardiac death, heart transplantation, or HF hospitalization” and hence excluded in the meta‐analysis of this outcome. QRS duration is found to be higher in nonresponders group mean difference −8.54 [−13.38, −3.70], *p* < .001; *I* ^2^: 70%, *p* = .07.

## 4 DISCUSSION

Fragmented QRS is associated with intraventricular dyssynchrony, higher Ts‐*SD*, positive Yu index, and independently predicts a nonresponse to CRT. Celikyurt et al. (2012 study was the leading cause of heterogeneity in the meta‐analysis. This is possibly due to this being the only study which shows higher incidence of ventricular dyssynchrony after implementation on CRT on patients without fragmented QRS on ECG. Interventricular and intraventricular dyssynchrony were highly prevalent in both patient groups with fragmented QRS and without fragmented QRS (64% vs. 75%, *p* = .44; 70% vs. 72.2%, *p* = .25 Respectively). Celikyurt et al. (2012 also had the broadest QRS duration (139.6 ± 15.9 ms) among variables analyzed for intraventricular dyssynchrony prediction; the others were mostly narrow except one study by Zhao et al. which did not specify but has a mean duration of 100.7 ± 8.5 ms.

It is interesting to note in a study by Celikyurt et al. (2012 that interventricular dyssynchrony was more prevalent in patients with fragmented QRS wave while intraventricular dyssynchrony was more prevalent in patients without fragmented QRS wave. The findings of this study contradict the previous findings and hypothesis on other studies that stated fragmented QRS and its underlying electrical remodeling on ventricular wall as a predisposing factor of ventricular dyssynchrony after CRT implantation The result of study by Celikyurt et al. (2012 is in accordance with a study by Yu et al. (2006 that stated that the majority of patients with dyssynchrony would be found to have narrow QRS complex; however, systolic asynchrony is the most common findings in patients with wide QRS complex (Yu et al., 2007). We found this finding to be in accordance with the findings in this meta‐analysis, in the terms that patients with fragmented QRS were over 20 times more likely to suffer from intraventricular asynchrony.

The findings of our analysis on the relation of fragmented QRS with CRT responders were supported by Varma (2009. It is shown in this study that the significance of fragmented QRS as a predictor of CRT responders stems from the findings that LV conduction delays and LV conductivity in patients with heart failure varied significantly with QRS configuration. It is found that LV conduction delays exceeding 100 ms were only found in 23% patients with narrow QRS configuration. The authors stated that these variations might affect CRT efficacy in patients with widened QRS configuration (Varma, 2009). Fragmented QRS causes heterogeneous ventricular activation along with dyssynchrony in patients with ischemic and non–ischemic cardiomyopathy, possibly impeding the efficacy of pacemaker (Basaran et al., 2011; Das et al., 2008). A higher number of leads with fQRS predict a nonresponse to CRT (Celikyurt et al., 2013). There are several factors such as left ventricular end‐diastolic diameter that may also affect nonresponsiveness to CRT; however, our meta‐analysis showed that the fragmented QRS is independent to other factors considered/found in the respective studies. In a study by Takenaka et al. (2019, QRS widening was shown to be related to myocardial viability. The authors stated that both myocardial viability and improved electrical dyssynchrony might be essential to predict a positive response to CRT (Takenaka et al., 2019).

The clinical implication of this study is that fQRS can be used as a marker for intraventricular dyssynchrony for both narrow and wide QRS complex. This may potentially identify patients that will benefit from CRT, although more study is needed. Fragmented QRS was also a predictor of nonresponse in patients receiving CRT; this interpretation is limited to those with wide QRS complex. Combined with other predictors, a scoring system can be developed to better identify patients that at risk of failing to receive benefits from CRT implantation. Hence, unnecessary CRT implantation can be avoided. The interpretation of fQRS, however, was known to have a poor inter‐ and intra‐observer variability in patients with cardiomyopathy according to a study (Vandenberk et al., 2018).

Limitation of this systematic review and meta‐analysis includes publication bias in which studies with positive results were more likely to be published and included in this systematic review. The pooled sample size is relatively small, and the definition of intraventricular dyssynchrony and nonresponse to CRT varied between studies. Future studies can address the issues of CRT implantation in those with fQRS and narrow QRS complex; the studies in this meta‐analysis only address that fQRS is associated with intraventricular dyssynchrony but not whether they will benefit from CRT implantation. This issue should be addressed in order to see whether CRT implantation is justifiable in HF with narrow fQRS.

## 5 CONCLUSION

Fragmented QRS is associated with intraventricular dyssynchrony and is independently associated with nonresponse to cardiac resynchronization therapy. Further studies need to be done to address whether intraventricular dyssynchrony detected by fQRS should proceed with CRT implantation.

## CONFLICT OF INTEREST

The authors declare that they have no conflict of interests.

## AUTHOR CONTRIBUTIONS

Raymond Pranata conceived and designed the study, interpreted the data, and drafted the manuscript. Alexander Edo Tondas and Rachel Vania performed a systematic search for the literature. Raymond Pranata and Emir Yonas extracted the data and assessed the risk of bias. Yoga Yuniadi critically revised the manuscript. Raymond Pranata performed the meta‐analysis in this study. All authors contributed to the writing of the manuscript.

## ETHICAL APPROVAL

This article does not contain any studies with human participants or animals performed by any of the authors, and thus, no ethical approval is required.
